# Incidence, Causes and Outcome of Obstructed Labor in Jimma University Specialized Hospital

**DOI:** 10.4314/ejhs.v20i3.69443

**Published:** 2010-11

**Authors:** Shimelis Fantu, Hailemariam Segni, Fessahaye Alemseged

**Affiliations:** 1 Department of Obstetrics and Gynecology, Jimma University, Jimma, Ethiopia; 2 Department of Epidemiology, Jimma University

**Keywords:** Obstructed labor, cephalo-pelvic disproportion, uterine rupture, Jimma

## Abstract

**Background:**

Obstructed labor is one of the common preventable causes of maternal and perinatal morbidity and mortality in developing countries. Africa has the highest maternal mortality in the world, estimated at an average of about 1,000 deaths per 100,000 live births. This study was conducted to assess the incidence, causes and outcome of obstructed labor in Jimma University Specialized Hospital.

**Methods:**

Hospital-based, cross-sectional study was conducted on all mothers who were admitted and delivered in the labor ward of Jimma University Specialized Hospital from November 1, 2008 to April 30, 2009. Data was collected using structured questionnaire and checklist, and then analyzed using SPSS for windows version 16.0.

**Results:**

The incidence of obstructed labor was 12.2%. Out of these 61.5% did not have antenatal care follow-up. Most of the cases, accounting for 145(81.0%), 160 (89.4%) and 170 (93.9%) were referred from health centers, visited the hospital after at least 12 hours of labor and came from a distance of more than 10 kilometers, respectively. The causes of obstructed labor were cephalo-pelvic disproportion in 121(67.6%) and malpresentation in 50 (27.9%) of the cases. The commonest maternal complications observed were uterine rupture in 55 (45.1%) and sepsis in 48 (39.3%) of the cases with complications. Forty-five point eight percent of fetuses were born alive and all had low first minute APGAR score.

**Conclusion:**

The incidence of obstructed labor was high with high rate of complications. The antenatal care follow-up practice was also found to be low. Improved antenatal care coverage, good referral system, and availing comprehensive obstetric care in nearby health institution are recommended to prevent obstructed labor and its complications.

## Introduction

Obstructed labor is an important cause of maternal death in communities in which childhood under nutrition and early marriage is common resulting in small pelvis, and in which there is no easy access to functioning health facilities with the capability of carrying out operative deliveries. Obstructed labor also causes significant maternal morbidity mainly due to infection and hemorrhage and in the long term leads to obstetric fistulae, skeletal and neurologic complications. Fetal death from asphyxia is also common (1).

There are different studies in developing countries showing incidences of obstructed labor varying from as low as 1.3% in a Sudan study to as high as 7% in a retrospective study done at Jimma University Specialized Hospital (JUSH) (2, 3, 4, 5).

The major cause of obstructed labor identified in different studies was Cephalo-pelvic disproportion being responsible for 80.6% in JUSH, 67% in a Nigerian study, and 41.1% in an Indian study (2, 3, 5). Several procedures are done to relieve the obstruction in obstructed labor. The major procedure done was cesarean delivery (C/S) which was done in 85% of cases in a Nigerian study and 63.3% of cases in Indian study (3, 5).

Complications observed in women with obstructed labor at studied areas were puerperal sepsis in 57% of cases in Nigeria to 12.5% in India and extension at time of surgery in 14% of cases in India. Maternal mortality from obstructed labor ranges from 32/1000 in Nigeria to 91/1000 in Jimma University Specialized Hospital. The perinatal mortality was 160/1000 in India, 294/1000 in Nigeria and 621/1000 in JUSH (2, 3, 4, 5).

It is customary to see lots of mothers coming to JUSH from its catchment areas, with obstructed labor, in which most of them have complications related to the illness and lose their perinates after carrying for nine months. A retrospective study was done ten years earlier on the problem in the same hospital. However, the study used retrospective data collection method which could have limitations on the quality of data and needs to be updated as it was done ten years back. This study was therefore conducted to determine incidence, causes and outcomes of obstructed labor in the hospital. This would help to have a broader and recent picture on the problem. It also helps to compare the findings with previous study done in the same hospital which could give a picture about the status of quality of health service care.

## Subjects and Methods

This hospital-based prospective cross-sectional study was conducted from November 1, 2008 to April 30, 2009 in Jimma University Specialized Hospital, which is found in Jimma City, South-western Ethiopia. It serves as the referral hospital for south-western part of the country and most of the laboring mothers come from rural areas where most deliveries are attended at home.

All mothers who were admitted to the labor ward with diagnosis of true labor during the six month study period were included in the study.

Data were collected using questionnaire and check list which contains socio-demographic characteristics of the patients, reported distance from the hospital, clinical features of obstructed labor, the mode of delivery and outcome on the mother and baby. Data were gathered by medical interns and residents in department of obstetrics and gynecology from patients' records and by interviewing the patients and physicians who were taking care of the patients during their stay in the ward. The patients were followed through their whole stay in the hospital so as to assess presence and development of complications.

Analysis was done using SPSS for windows version 16.0 to describe variables and assess associations. Obstructed labor was operationally defined as failure of the presenting part to descend in birth canal despite adequate uterine contractions for mechanical reasons. The diagnosis of obstructed labor was made by gynecology residents working in the hospital. The term destructive operation was operationally defined to refer to procedure done on dead fetus to effect vaginal delivery.

Ethical clearance was obtained from Jimma University medical sciences faculty ethical review committee and permission to conduct the study was obtained from JUSH. Verbal consent was obtained from the study subjects and the right of the respondents to withdraw or not to participate was respected. Additionally, names of participants were not used in the study and confidentiality of the patient information was maintained

## Results

During the 6 months study period, there were a total of 1468 deliveries of which 179 were diagnosed to have obstructed labor (12.2%). Fifteen (8.4%) of the obstructed labor cases were teenagers and the majority, 110 (61.5%) in the age group of 20–29 years. All of the mothers with obstructed labor were married and majority of them were Muslim by religion, Oromo in Ethnicity and Para II–IV accounting for 133(74.3%), 143(79.9%) and 75(41.9%), respectively (Table 1).

**Table 1:** Background characteristics of obstructed labor cases, JUSH, Nov 2008–April 2009.

Background information		Number of cases (N=179)	Percent
Age			
	≤19	15	8.4
	20–29	110	61.5
	30–34	30	16.8
	≥35	24	13.4
Religion			
	Muslim	133	74.3
	Christian	46	25.7
Ethnicity			
	Oromo	143	79.9
	Amhara	15	8.4
	Dawro	8	4.5
	Keffa	7	3.9
	Others	6	3.4
Parity			
	Primipara	61	34.1%
	Para II–IV	75	41.9%
	Para ≥V	43	24.0%
Total		179	100.0%

One hundred ten (61.5%) of the obstructed labor cases did not have any ANC follow up while the rest had at least one visit. Most of the cases 145(81.0%), 160 (89.4%) and 170 (93.9%) were referred from health centers, visited JUSH after at least 12 hours of labor and came from a distance of more than 10 kilometers, respectively (Table 2).

**Table 2:** Distribution of cases with obstructed labor in relation utilization and access to health services, JUSH, Nov 2008–April 2009.

Health service utilization	Number of cases (n=179)	Percent
ANC follow-up		
Had at least one follow-up	69	38.5
Had no follow-up	110	61.5
Source of referral		
Self	16	8.9
Hospital	18	10.1
Health center	145	81.0
Duration of labor before visiting JUSH		
<12	19	10.6
12–24	87	48.6
>24	73	40.8
Distance from JUSH		
<10KM	9	5.0
10–50KM	83	46.4
51–100KM	47	26.3
>100KM	40	22.3

The duration of labor before arrival to JUSH was assessed for its association with age, parity, ANC follow-up and distance from JUSH. The only variable found to have statistically significant association was distance from JUSH (Table 4).

**Table 4:** Factors associated with delayed visit to JUSH among obstructed labor cases, Nov 2008 – Apr 2009.

Independent variables	Categories	Duration of labor before arrival to JUSH Frequency (%)		COR (95%CI)	AOR(95% CI)
				
		⇐=24hours	>24hours		
Age	⇐=19	5 (33.3)	10 (66.7)	2.0(0.5–7.6)	1.9(0.4–10.2)
	20–29	71(64.5)	39 (35.5)	0.6(0.2–1.3)	0.6(0.2–1.9)
	30–34	18 (60.0)	12 (40.0)	0.7(0.2–2.0)	0.5(0.2–1.6)
	>=35	12(50.0)	12(50.0)	1	1
Parity	Primipara	32(52.5)	29(47.5)	1.0(0.4–2.1)	1.4(0.5–3.7)
	Para II–IV	52(69.3)	23(30.7)	0.5(0.2–1.0)	0.6(0.2–1.4)
	Para V or more	22(51.2)	21(48.8)	1	1
ANC follow-up	Had at least one follow-up	52(75.4)	17(24.6)	0.3(0.2–0.6)	0.3(0.1–0.6)
	Had no follow-up	54(49.1)	56(50.9)	1	1
Distance from JUSH	<10Kms	10(90.9)	1(9.1)	1	1
	10–50Kms	47(56.0)	37(44.0)	7.9(1.0–64.3)	13.1(1.4–120.8)
	>50Kms	49(58.3)	35(41.7)	7.1(0.9–58.4)	11.2(1.2–102.4)

**Figure 1:**
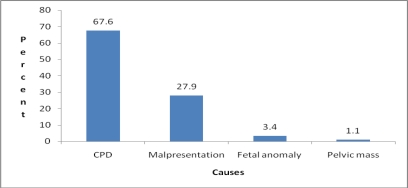
Distribution of cases by cause of obstructed labor, JUSH, Nov 2008–April 2009.

With regard to causes of obstructed labor, cephalopelvic disproportion (CPD) was the main cause in 121 (67.6%) followed by malpresentation in 50 (27.9%) of the cases (Figure 1). No case with scared uterus was seen.

**Figure 2:**
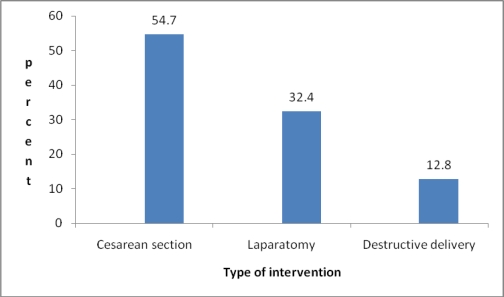
Interventions done in cases of obstructed labor, JUSH, Nov 2008–April 2009.

The commonest type intervention was C/S in 98 (54.7%) of the patients, followed by laparatomy in 58 (32.4%) and destructive delivery (craniotomy and evisceration) in 23 (12.8%) (Figure 2). Laparatomy was done for three cases of post partum hemorrhage (PPH) after C/S and for one case after destructive delivery. The indications for hysterectomy were uterine rupture and PPH.

Uterine rupture was the commonest complication of obstructed labor which occurred in 55 (45.1%) followed by sepsis in 48 (39.3%) of the cases. Bladder rupture was diagnosed intra operatively in three patients (2.6 %) (Table 3). Twenty seven cases (15.1%) had combination of complications.

**Table 3:** Distribution of complications in cases of obstructed labor, JUSH, Nov 2008–April 2009.

Complications	Number of cases	Percent
Uterine rupture	55	45.1
Sepsis	48	39.3
PPH	10	8.2
VVF	5	4.1
Bladder rupture	3	2.6
Wound dehiscence	1	0.8
**Total**	**122**	**100.0**

Age, parity, history of ANC follow-up and duration of labor were assessed for association with presence of at least one complication of obstructed labor; none of the factors were found to have statistically significant association (Table 5).

**Table 5:** Factors associated with presence of at least one obstetric complication among obstructed labor cases, Nov 2008 – Apr 2009.

Independent variables	Categories	Presence of at least one complication* Frequency (%)		COR (95% CI)	AOR(95% CI)
				
		No	Yes		
Age	⇐=19	10(66.7)	5(33.3)	0.4(0.1–1.6)	2.1(0.4–10.6)
	20–29	60(54.5)	50(45.5)	0.7(0.3–1.7)	1.7(0.6–5.0)
	30–34	8(26.7)	22(73.3)	2.3(0.7–7.3)	3.0(0.9–10.0)
	>=35	11(45.8)	13(54.2)	1	1
Parity	Primipara	46(75.4)	15(24.6)	0.2(0.1–0.4)	0.2(0.1–0.4)
	Para II–IV	29(38.7)	46(61.3)	0.8(0.4–1.7)	0.8(0.3–1.9)
	Para V or more	14(32.6)	29(67.4)	1	1
ANC follow-up	Had at least one follow-up	41(59.4)	28(40.6)	1.9(1.0–3.5)	0.7(0.3–1.3)
	Had no follow-up	48(43.6)	62(56.4)	1	1
Duration of labor before arrival to JUSH	<24 hours	54(50.9)	52(49.1)	1	1
	>=24 hours	35(47.9)	38(52.1)	1.1(0.6–2.1)	1.2(0.6–2.5)

* The complications considered were: PPH, sepsis, uterine rupture, bladder rupture, and fistula

Eighty two (45.8%) of the fetuses were born alive and all of them had low APGAR score in the first minute. The fifth minute APGAR score was normal for 62 (75.6%) of the live born. The perinatal mortality rate was 66.1 per 1000 births. The weight of the 153 (85.5%) of the newborns was in the normal range (2500–3999 grams), whereas 16 (8.9%) of them were macrosomic (>4000 grams) and the other 10 (5.6%) had low birth weight (1500–2499 grams).

## Discussion

This study had tried to look at incidence, causes and outcomes of obstructed labor. It showed the burden that it could bring to the health service, the community and the country in general. One limitation of the study is it has not addressed maternal mortality which is an important indicator though other outcomes like complications on the mother and on the fetus including perinatal mortality are assessed.

This study showed a high incidence of obstructed labor (12.2%) compared to the previous studies done in this hospital and other African countries (2, 3, 4, 5). This might be due to, the Hospital is a referral hospital covering wide catchment area and most of the patients referred were already complicated.

Sixty-two percent of the obstructed labor cases did not have any ANC follow-up; similarly in other studies the unbooked ladies are also more affected by obstructed labor (2, 3). Even though ANC is a poor measure to prevent pregnancy and delivery complications, it could be good time to discuss about preparation for delivery and to go to the proper health institution earlier. Most of the cases had labor for more than twelve hours and 30.7% of them came with uterine rupture.

Cephalo-pelvic disproportion was the major cause of obstructed labor (67.6%), which is comparable to the study done in Nigeria (3), but lower than the previous study undertaken in this hospital (2). The fact that the weight of most fetuses were in the range of 2500–3999 grams signifies as contracted pelvis or malposition was the reason for the CPD than fetal size.

Cesarean section was the main way of delivery (54.7%), which is lower than the study done in Nigeria and India (3, 6), but it is more than the previous study of this hospital (2). Wound infection is the commonest complication of cesarean section (35.7%) which may be due to failure to use prophylactic antibiotic properly. Destructive delivery was the least frequent mode of delivery compared to the other studies (2, 6, 7) which could be explained by the high number of cases of uterine rupture on arrival on whom destructive delivery is contraindicated.

Uterine rupture was the commonest complication of obstructed labor followed by sepsis in this study as uterine rupture is a well known contributor of maternal hemorrhage and sepsis, which are major causes of maternal mortality and morbidity. But in the Nigerian study, sepsis was commonest complication (3). This may be due to their late arrival to this hospital after onset of labor as compared to the latter study; most cases arrived after twelve hours of labor. The other reason could be the higher proportion of multiparous ladies in this study (65.9%) who are at increased risk of uterine rupture.

The study also showed obstructed labor to be one of the major causes of poor perinatal outcome with low first minute APGAR score, and perinatal death (66.1 per 1000 births). This is a bit higher compared to the previous figure of this hospital (2), but it is too low than other studies made in Nigeria and India (3, 5).

In conclusion, this study revealed high incidence of obstructed labor and its complications as well as low ANC follow-up and delayed arrival to hospital. In order to alleviate these problems, the Ministry of Health and other responsible bodies need to exert efforts to increase the ANC follow-up coverage so that high risk mothers could be detected, improve functioning of health centers and the referral system as well as scaling up of the transportation system.
